# Tau acetylated PHF6 reactive T cells presented by HLA‐DRB1*04:04 in Alzheimer's Disease

**DOI:** 10.1002/alz70855_103896

**Published:** 2025-12-24

**Authors:** Guo Luo, Jing Zhang, Emmanuel Mignot

**Affiliations:** ^1^ Stanford University School of Medicine, Palo Alto, CA, USA

## Abstract

**Background:**

Neurofibrillary tangles (NFTs) are hallmarks of Alzheimer's disease (AD) and formed by aggregation of the microtube‐associated protein tau that undergoes many modifications, including acetylation on lysine (K) in tau. A piece of tau consisting of six amino acid residues, paired helical filament (PHF)6 (VQIVYK), plays a predominant role in tau aggregation. Merging evidence supports adaptive immune system plays an important role. However, the pieces of antigens, the types and functions of T cells, and HLA types associated with AD remain largely unknown.

**Methods:**

We analyzed HLA associations in ∼176,000 individuals with PD or AD versus controls across ancestry groups. We also compared postmortem brain density of NFTs and amyloid plaques in brain, tau and Aβ42 levels in cerebrospinal fluid (CSF) of ∼8,000 individuals (controls and AD), and examined association of HLA in ∼2,500 patient with pathologically demonstrated Lewy Body Dementia. This was followed by HLA binding, tetramer T cell studies and TCR activation by tau PHF6 in Jurkat cell.

**Results:**

We found that HLA‐DRB1*04:04 has protective effect in AD. We detected clonally expanded T cells recognizing tau, especially modified PHF6 with acetylation at lysine 311 (PHF6 aK311) when presented by DRB1*04:04, and the phenotypes of these T cells are distinct in AD patients versus cognitively healthy controls. Further, we identified different TCR clones from both AD patients and healthy controls only activated by tau PHF6 aK311 presented by DRB1*04:04.

**Conclusion:**

An HLA‐DRB1*04‐mediated adaptive immune response against tau PHF6 aK311 decreases AD risk, offering the possibility of new therapeutic avenues.

